# Performance of Large Language Models on a Neurology Board–Style Examination

**DOI:** 10.1001/jamanetworkopen.2023.46721

**Published:** 2023-12-07

**Authors:** Marc Cicero Schubert, Wolfgang Wick, Varun Venkataramani

**Affiliations:** 1 Neurology Clinic and National Center for Tumor Diseases, University Hospital Heidelberg, Heidelberg, Germany; 2 Clinical Cooperation Unit Neurooncology, German Cancer Consortium, German Cancer Research Center, Heidelberg, Germany

## Abstract

**Importance:**

Recent advancements in large language models (LLMs) have shown potential in a wide array of applications, including health care. While LLMs showed heterogeneous results across specialized medical board examinations, the performance of these models in neurology board examinations remains unexplored.

**Objective:**

To assess the performance of LLMs on neurology board–style examinations.

**Design, Setting, and Participants:**

This cross-sectional study was conducted between May 17 and May 31, 2023. The evaluation utilized a question bank approved by the American Board of Psychiatry and Neurology and was validated with a small question cohort by the European Board for Neurology. All questions were categorized into lower-order (recall, understanding) and higher-order (apply, analyze, synthesize) questions based on the Bloom taxonomy for learning and assessment. Performance by LLM ChatGPT versions 3.5 (LLM 1) and 4 (LLM 2) was assessed in relation to overall scores, question type, and topics, along with the confidence level and reproducibility of answers.

**Main Outcomes and Measures:**

Overall percentage scores of 2 LLMs.

**Results:**

LLM 2 significantly outperformed LLM 1 by correctly answering 1662 of 1956 questions (85.0%) vs 1306 questions (66.8%) for LLM 1. Notably, LLM 2’s performance was greater than the mean human score of 73.8%, effectively achieving near-passing and passing grades in the neurology board examination. LLM 2 outperformed human users in behavioral, cognitive, and psychological–related questions and demonstrated superior performance to LLM 1 in 6 categories. Both LLMs performed better on lower-order than higher-order questions, with LLM 2 excelling in both lower-order and higher-order questions. Both models consistently used confident language, even when providing incorrect answers. Reproducible answers of both LLMs were associated with a higher percentage of correct answers than inconsistent answers.

**Conclusions and Relevance:**

Despite the absence of neurology-specific training, LLM 2 demonstrated commendable performance, whereas LLM 1 performed slightly below the human average. While higher-order cognitive tasks were more challenging for both models, LLM 2’s results were equivalent to passing grades in specialized neurology examinations. These findings suggest that LLMs could have significant applications in clinical neurology and health care with further refinements.

## Key Points

**Question:**

What is the performance of large language models on neurology board–style examinations?

**Findings:**

In this cross-sectional study, a newer version of the large language model significantly outperformed the mean human score when given questions from a question bank approved by the American Board of Psychiatry and Neurology, answering 85.0% of questions correctly compared with the mean human score of 73.8%, while the older model scored below the human average (66.8%). Both models used confident or very confident language, even when incorrect.

**Meaning:**

These findings suggest that with further refinements, large language models could have significant applications in clinical neurology.

## Teaser

This cross-sectional study assesses the performance of large language models (LLMs) on neurology board–style examinations.

## Introduction

Deep learning algorithms have been investigated in neurology for a variety of tasks, such as neurologic diagnosis, prognosis, and treatment.^1,2^ However, the role and potential application of large language models (LLMs) in neurology have been unexplored. The recent emergence of powerful transformer-based artificial intelligence models^3,4^ provides a new avenue for exploring their implications in the field of neurology. These LLMs undergo training using expansive data sets, encompassing more than 45 terabytes of information. This rigorous training process equips them to recognize patterns and associations among words, which, in turn, empowers them to produce responses that are both contextually accurate and logically consistent.^5^ While the newer model performed better in a variety of examinations,^5^ the older model remains faster in processing tasks. The application of these models in specialized medical examinations has been tested to some extent. The older model showed near-pass performance in the United States Medical Licensing Examination (USMLE),^6,7^ while it failed to pass the ophthalmology board examination.^8^ Two recent reports showed slightly deviating results on neurosurgery board–style examinations, with 1 report claiming a near-pass with the older model and the other showing an approximately 10% lower performance. The older model achieved a near-pass in radiology board–like examinations,^9^ while the new model successfully passed neurosurgery board–like examinations.^10^ In contrast, neurology board–like examinations present a different set of challenges. Compared with radiology, ophthalmology, or neurosurgery, the questions in neurology board examinations often present complex narratives with subtle diagnostic clues that require a nuanced understanding of neuroanatomy, neuropathology, and neurophysiology. The candidate is expected to navigate through these complex narratives, extracting relevant data, and synthesizing this information into a coherent diagnostic hypothesis and subsequent therapeutic decisions. Written board examinations, designed to test a broad range of neurology topics, are common in the United States, Canada, and Europe. These examinations typically use multiple-choice questions, a format also adopted in the United States by the American Board of Psychiatry and Neurology (ABPN)^11^ and in Europe by the European Board of Neurology (UEMS-EBN).^12^

In this cross-sectional study, our objective was to evaluate the performance of these 2 models in comparison with human performance in neurology board–like written examinations. We used the context of neurology board–like written examinations as a representative example to scrutinize the complex reasoning abilities and the capacity of LLMs to navigate intricate medical cases, thereby illuminating their potential in more sophisticated, real-world clinical applications. Our ultimate aim was not only to determine their accuracy and reliability in this specialized context but also to characterize their strengths and limitations. As LLMs continue to evolve, understanding their potential contributions and challenges in medical examinations could pave the way for future applications in neurology and neurology education.

## Methods

### Standard Protocol Approvals, Registrations, and Patient Consents

This study did not involve human participants or patient data, so it was exempt from institutional review board approval. This report follows the Strengthening the Reporting of Observational Studies in Epidemiology (STROBE) reporting guideline for observational studies. This exploratory prospective study was performed from May 17 to May 31, 2023.

### Multiple-Choice Question Selection and Classification

A question bank resembling neurology board questions consisting of 2036 questions^13^ was used for the evaluation of 2 LLMs: GPT-3.5 (LLM 1) and GPT-4 (LLM 2). Questions including videos or images as well as questions that were based on preceding questions were excluded in this study (80 questions excluded; 1956 included). This question bank is approved by the ABPN as part of a self-assessment program and can be used as a tool for certified medical education.^13^ Questions were in single best answer, multiple-choice format with 3, 4, or 5 distractors and 1 correct answer. To validate the results from this question bank, open-book sample questions from 2022 from the European Board of Neurology were used (19 questions). These questions are either behind a paywall (in the case of the question bank) or published after 2021 and therefore out-of-training data for both LLMs. Memorization analyses^14,15^ were performed to exclude the possibility that test questions were in-training data for the models (eMethods in Supplement 1).

Questions were then classified by type using principles of the Bloom taxonomy for learning and assessment as testing either lower-order (remembering, basic understanding) or higher-order (applying, analyzing, or evaluating) thinking.^16,17^ In 400 randomly sampled questions, we both let LLM 2 and the investigators evaluate whether the questions were in the lower-order or higher-order category, and the investigators discussed cases of incongruencies. LLM 2 classified in accordance with the investigators in 84.5% of cases (338 of 400 questions), LLM 1 did so in 87.0% of cases (348 of 400 questions). Question length was assessed by counting the characters of the question.

The questions can be further categorized according to 26 topics in the field of neurology that are listed in the Table. The percentage of users who answered correctly per individual question was available from the test portal while this information was not available for the sample questions from the EBN.

**Table.** Performance of LLMs and Question Bank Users by Question Type and Topic

Question type	Questions, No.	Human correct, mean %	Correct answers, No. (%)		Adjusted *P* value^a^		
			LLM 1	LLM 2	LLM 1 vs human	LLM 2 vs human	LLM 1 vs LLM 2
All questions	1956	73.8 (17.9)	1306 (66.8)	1662 (85.0)	<.001	<.001	<.001
Order of thinking							
Higher	1063	73.9 (17.9)	667 (62.7)	872 (82.0)	<.001	<.001	<.001
Lower	893	73.6 (17.8)	639 (71.6)	790 (88.5)	.73	<.001	<.001
Category							
Basic neuroscience	128	74.1 (16.7)	83 (64.8)	109 (85.2)	>.99	>.99	.008
Behavioral, cognitive, psychological	482	76.0 (17.8)	362 (75.1)	433 (89.8)	>.99	<.001	<.001
Cerebrovascular	113	77.7 (16.7)	77 (68.1)	93 (82.3)	>.99	>.99	.54
Child neurology	73	69.8 (20.7)	48 (65.8)	57 (78.1)	>.99	>.99	>.99
Congenital	20	74.3 (11.2)	14 (70.0)	18 (90.0)	>.99	>.99	>.99
Cranial nerves	46	70.0 (17.2)	24 (52.2)	34 (73.9)	>.99	>.99	>.99
Critical care	28	71.3 (19.5)	15 (53.6)	25 (89.3)	>.99	>.99	.20
Demyelinating disorders	49	81.9 (15.8)	37 (75.5)	45 (91.8)	>.99	>.99	>.99
Epilepsy, seizures	55	73.7 (17.0)	34 (61.8)	39 (70.9)	>.99	>.99	>.99
Ethics	5	84.8 (11.7)	2 (40.0)	4 (80.0)	>.99	>.99	>.99
Genetic	20	70.4 (15.8)	16 (80.0)	17 (85.0)	>.99	>.99	>.99
Headache	59	74.0 (19.5)	40 (67.8)	50 (84.7)	>.99	>.99	>.99
Imaging or diagnostic studies	10	65.4 (24.5)	5 (50.0)	9 (90.0)	>.99	>.99	>.99
Movement disorders	91	75.1 (14.7)	54 (59.3)	79 (86.8)	>.99	>.99	.002
Neuro-ophthalmology	46	72.4 (15.9)	29 (63.0)	40 (87.0)	>.99	>.99	.42
Neuro-otology	42	66.9 (18.1)	20 (47.6)	31 (73.8)	>.99	>.99	.66
Neuroinfectious disease	30	68.1 (19.5)	16 (53.3)	25 (83.3)	>.99	>.99	.69
Neurologic complications of systemic disease	42	71.8 (22.2)	21 (50)	31 (73.8)	>.99	>.99	>.99
Neuromuscular	184	68.9 (19.2)	120 (65.2)	145 (78.8)	>.99	>.99	.14
Neurotoxicology, nutrition, metabolic	93	70.4 (17.8)	63 (67.7)	82 (88.2)	>.99	.10	.04
Oncology	72	70.0 (18.3)	41 (56.9)	62 (86.1)	>.99	.71	.006
Pain	65	78.8 (17.5)	42 (64.6)	58 (89.2)	>.99	>.99	.05
Pharmacology	72	74.0 (15.6)	48 (66.7)	60 (83.3)	>.99	>.99	.89
Pregnancy	19	69.1 (16.9)	14 (73.7)	15 (78.9)	>.99	>.99	>.99
Sleep	92	77.4 (15.1)	67 (72.8)	82 (89.1)	>.99	>.99	.22
Trauma	20	74.2 (17.0)	14 (70.0)	19 (95.0)	>.99	>.99	>.99

Abbreviation: LLM, large language model.

^a^ The χ^2^ test was used to calculate *P* values. *P* values were adjusted for multiple testing using the Bonferroni correction.

### LLMs

LLM 1 and 2 were used via the application programming interface (API). These are 2 commonly used LLMs.^3,5^ At the time of this study, we did not have access to other powerful closed-source models.^18,19^ LLM 1 and LLM 2 were pretrained on more than 45 terabytes of text data, including a substantial portion of internet websites, books, and articles. The investigators did not perform any additional neurology-specific fine-tuning of the model. In this study, we used server-contained language models that were trained up to September 2021. The used models do not have the ability to search the internet or external databases.

### Data Collection and Assessment

Each multiple-choice stem along with its answer choices was provided to the models via its API together with the following prompt:

“You are a medical doctor and are taking the neurology board exam. The board exam consists of multiple choice questions.

All output that you give must be in JSON format.

- Return the answer letter

- Give an explanation

- Rate your own confidence in your answer based on a Likert scale that has the following grades: 1 = no confidence [stating it does not know]; 2 = little confidence [ie, maybe]; 3 = some confidence; 4 = confidence [ie, likely]; 5 = high confidence [stating answer and explanation without doubt])

- Classify the question into the following two categories: 1. lower order questions that probe remembering and basic understanding, and 2. higher order question where knowledge needs to be applied, analysis capabilities are examined, or evaluation is needed. (return “Higher” or “Lower”)

- Rate the confidence of your classification into these categories based on the Likert scale that has the following grades1 = no confidence [stating it does not know]; 2 = little confidence [ie, maybe]; 3 = some confidence; 4 = confidence [ie, likely]; 5 = high confidence [stating answer and explanation without doubt])

Your output must look like the following:

{”answerletter”:…,”reasoning”:…,”confidence_answer_likert”:…,”classification”:…,” confidence_classification_likert”:…”

All answer choices and responses were recorded. A passing score was considered 70% or higher on this neurology board–style examination without images to approximate the written examination from the ABPN and the EBN. The question bank uses 70% as passing threshold to gain credits for certified medical education (CME) points. The Royal College examination in Canada considers 70% or greater on all written components a passing score. There, questions undergo psychometric validation, with removal of questions found not discriminatory or too difficult, which was not performed. The ABPN and EBN examinations use criterion-referenced scoring, which was not used.

### Reproducibility of Answers

For the reproducibility analyses, 100 questions were answered by both models with 50 independent queries probing the principle of self-consistency^20^ and the percentage of each question was recorded. Then, answers with high reproducibility (defined as more than 75% of all queries answered with the same answer) were compared with answers without high reproducibility.

### High-Dimensional Analysis of Question Representations by the LLMs

For the high-dimensional analysis of question representations, the embeddings of these questions were analyzed. These numeric vector representations encompass the semantic and contextual essence of the tokens (in this context, the questions) processed by the model.^21^ The source of these embeddings is the model parameters or weights, which are used to code and decode the texts for input and output. A dimensionality reduction of the embeddings was performed with a t-distributed stochastic neighbor embedding (tSNE) analysis,^22^ and clusters were subsequently examined. Similarity between question and answer embeddings was compared calculating cosine similarity.

### Statistical Analysis

First, the overall performance was evaluated. Next, we compared the performance across different types of questions (namely, lower and higher order) using a single-variable analysis approach (employing the χ^2^ test). We also executed a subgroup analysis for various subclasses of higher-order thinking questions and the 26 topics, in which we utilized the χ^2^ test for multiple comparisons with a Bonferroni correction. Given that the models had a probabilistic chance of correctly answering each question, we utilized a guessing correction formula^23^ to glean further understanding: it is computed by subtracting the ratio of the number of incorrect responses to the total number of choices minus one from the number of correct responses: *n*_correct_ − (*n*_wrong_ / [*k*_options_ − 1).

We contrasted the confidence level of responses between correct and incorrect answers with the Mann-Whitney *U* test after testing for normality using the Shapiro-Wilk test. For the correlation analysis between human performance and model performance, human quartiles were converted to numeric values (1-4, with 1 corresponding to the 25% of questions that had the highest human scores and 4 corresponding to the 25% of questions with the lowest human scores). A *P* value of less than .05 was deemed indicative of a significant difference. All these statistical examinations were carried out in R version 4.0.5 (R Project for Statistical Computing).

## Results

### Overall Performance

First, we examined the proficiency of LLM 1 and LLM 2 against a question bank set. LLM 2 had an 85.0% accuracy level (1662 correct responses of 1956 questions), superseding LLM 1, which managed a 66.8% accuracy level (1306 correct responses of 1956 questions). When adjusting for random guessing, LLM 2 yielded an 80.9% score (1583 of 1956), as opposed to LLM 1’s 57.8% score (1131 of 1956). In comparison with the average user of the testing platform (73.8%), LLM 2’s performance was superior (*P* < .001), whereas LLM 1 underperformed (*P* < .001) (Table).

To corroborate these results, we also investigated the performance based on openly available sample questions from the EBN for its board examination. Here, LLM 2 correctly responded in 73.7% of the questions (14 of 19 questions), while LLM 1 only gave a correct response in 52.6% of the questions (10 of 19 questions) (*P* = .31) (eTable 1 in Supplement 1).

### Performance by Question Type

Upon analyzing the performance based on question type, both models excelled in lower-order questions (LLM 1: 639 of 893 [71.6%]; LLM 2: 790 of 893 [88.5%]) compared with higher-order questions (LLM 1: 667 of 1063 [62.7%]; LLM 2: 872 of 1063 [82.0%]) (*P* < .001) (Table). In the context of lower-order questions, LLM 1’s performance was akin to human users’ performance (73.6%) (*P* = .73). However, LLM 1 lagged in answering higher-order questions vs human users (73.9%) (*P* < .001), as exhibited in the Table. In both lower and higher-order questions, LLM 2 surpassed LLM 1 and human users (*P* < .001) (Table).

**Figure 1.**
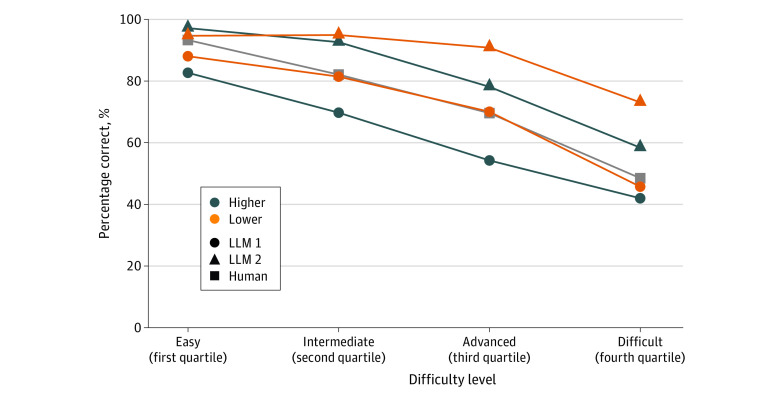
Performance of Large Language Models (LLMs) by Difficulty Level Difficulty was assessed based on the percentage of human users who answered correctly.

eFigures 1 to 4 in Supplement 1 provide examples of questions, both correctly and incorrectly answered by LLM 2, categorized into lower- and higher-order categories. Interestingly, when segregating the questions into quartiles according to the average performance of human users (ie, easy, intermediate, advanced, and difficult) a correlation between the performance of LLM 1, LLM 2, and the average human user (*R* = 0.84, *P* < .001). This correlation potentially suggests shared difficulties faced by humans and these LLMs, as depicted in Figure 1 and eTable 2 in Supplement 1.

### Performance by Topic

**Figure 2.**
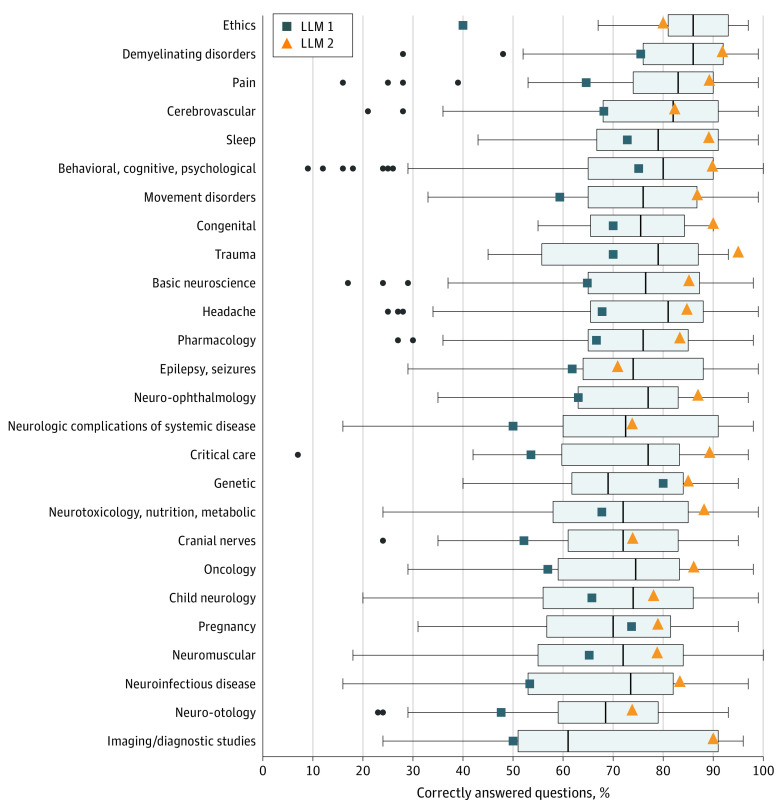
Percentage of Correctly Answered Questions per Topic Boxplots illustrate human user score distribution, with the black line indicating the median, the edges of the boxes indicating first and third quartiles, and the whiskers indicating the largest and smallest value no further than 1.5 × IQR from the lower and upper edges. Dots indicate outliers. LLM indicates large language model.

A comparative evaluation of LLM 1, LLM 2, and the average user performance across various topics was carried out (Figure 2). In the behavioral, cognitive, psychological category, LLM 2 outperformed both LLM 1 and average test bank users (LLM 2: 433 of 482 [89.8%]; LLM 2: 362 of 482 [75.1%]; human users: 76.0%; *P* < .001). LLM 2 also exhibited superior performance in topics such as basic neuroscience, movement disorders, neurotoxicology, nutrition, metabolic, oncology, and pain compared with LLM 1, whereas its performance aligned with the human user average (Table, Figure 2).

To identify any topic-specific strengths or weaknesses displayed by each model, we analyzed their performance in topics that contained more than 50 questions. Notably, LLM 1 did not display any significant performance variation across topics. In contrast, LLM 2 showed significantly enhanced performance in answering questions related to behavioral, cognitive and psychological categories (433 of 482 [89.8%]) compared with its performance on questions concerning epilepsy and seizures (39 of 55 [70.9%]) (*P* = .008) and neuromuscular topics (145 of 184 [78.8%]) (*P* = .02).

### Level of Confidence

Both models consistently responded to multiple-choice questions using confident or highly confident language (100%, 400 of 400 questions evaluated by investigators). Self-assessment of confidence expressed by LLM 1 and LLM 2 in their answers showed a small difference between incorrect and correct responses (mean [SD] Likert score for LLM 1, 4.69 [0.46] vs 4.79 [0.41]; *P* < .001; for LLM 2, 4.77 [0.45] vs 4.93 [0.30]; *P* < .001). Incorrect LLM 2 and LLM 1 answers were all subjectively assessed by the models as expressing confidence or high confidence (Likert score 4 or 5 for LLM 2: 292 of 294 [99.3%]; for LLM 1: 650 of 650 [100%]) (eFigure 5 in Supplement 1). When prompted with the correct answer after an incorrect response, both models responded by apologizing and agreeing with the provided correct answer in all cases.

### Reproducibility of Responses

Reproducibility analyses revealed that highly reproducible answers were more likely to be answered correctly than inconsistent answers by LLM 1 (66 of 88 [75.0%] vs 5 of 13 [38.5%]; *P* = .02), potentially indicating another marker of confidence of LLMs that might be leveraged to filter out invalid responses. The same observation was made with LLM 2, with 78 of 96 correct answers (81.3%) in those with high reproducibility vs 1 of 4 (25.0%) in answers with low reproducibility (*P* = .04).

### Characteristics of Questions Using High-Dimensional Representation Analysis of Question Embeddings

We identified an association of question word length and the ability to answer questions correctly in both models, with incorrectly answered questions being longer on average (eFigure 6 in Supplement 1). This was not found in human users, but instead a weak positive correlation between question length and correct answers was observed (*R* = 0.074; *P* = .001) (eFigure 6 in Supplement 1). When analyzing the high-dimensional representation of correctly and incorrectly answered questions, no pattern into distinct clusters was observed (eFigure 7 in Supplement 1).

To investigate whether the models use the similarity of question and answers in the multidimensional embedding space to select their answer, similarity between the question embedding and each answer embeddings was compared. It was found that in 28.3% of questions (476 of 1681), the correct answer was the closest in the multidimensional embedding space. Accordingly, the LLMs labeled the most similar answer as correct in more than 30% of cases (LLM 2: 513 of 1681 [30.5%]; *P* = .17; LLM 2: 524 of 1681 [31.1%]; *P* = .08), indicating that the distance between question and answer did not significantly affect the models’ answer choice.

## Discussion

The notable progress achieved by the 2 LLMs studied has substantially enhanced the potential of these models across a wide range of applications.^24,25,26^ Despite being extensively pretrained on vast data sets and offering promising possibilities within the health care sector, their specific application in neurology remains relatively uncharted territory. The efficacy of these models in handling specialized neurology knowledge also remained indeterminate until this study.

This exploratory research revealed LLM 2’s proficiency in completing a neurology board–like examination, a task LLM 1 was unable to accomplish. This finding underscores the rapid and significant evolution of LLMs. Our results suggest that LLM 2 would be able to pass a neurology board–like examination, whereas LLM 1’s performance falls short of passing such a specialized examination.

Despite their strengths, both models demonstrated weaker performance in tasks requiring higher-order thinking compared with questions requiring only lower-order thinking.^16^ In comparison with its performance on the USMLE Step Examinations,^6,27,28^ where it did not exceed a 65% accuracy, LLM 1 scored surprisingly well in this more specialized examination.

As these models are trained to identify patterns and relationships among words in their training data, they can struggle in situations requiring a deeper understanding of context or specialized technical language. This limitation is crucial for neurologists to bear in mind, particularly with LLMs now being incorporated into popular search engines and readily accessible to the public.^29^ It is important to note that the models have been changing over time.^30^ Therefore, for models to be reliably used in the medical context, it would be important to use models that remain stable and are continuously tested when updated.

Interestingly, both models exhibited confident language when answering questions, even when their responses were incorrect. This trait is a recognized limitation of LLMs^31,32^ and originates from the training objective of these models, which is to predict the most likely sequence of words following an input. This characteristic, coupled with the model’s inclination to generate plausible, convincing, and human-like responses, can potentially mislead individuals relying solely on it for information.^33,34^ However, the model was able to partially differentiate its own confidence level, as there were slight but important differences between correct and incorrect answers, although the values on the Likert scale predominantly are between confident and highly confident. Furthermore, we identified that reproducible answers are correlated with correctness and might serve as an intrinsic, surrogate marker of confidence defined by the output of the LLM.

### Limitations

This study has some limitations. The questions used were not official ABPN or EBN board examination questions due to their confidential and regulated nature. Additionally, image-based questions were not included because LLM 1 and versions of LLM 2 at the time of the study were not equipped to process these. Furthermore, the passing grade was an approximation based on the threshold by the ABPN for approving of points for CME. The limited number of questions in each subgroup in this exploratory study also reduced the power of subgroup analyses. We only included these 2 models in this assessment as other similarly powerful closed-source models were not available to us at the time of this study. The absence of significant differences between the 2 models in the EBN question bank should be considered in the context of limited number of questions in this cohort.

In addition, the level of education of the human scorers is not exactly known and should be considered when interpreting comparisons between the models and the users scores. Additionally, human scores could be overestimated due to users that repeat question banks for practice. Therefore, it is important to also consider the absolute scores from the models.

## Conclusions

In conclusion, this study underscored the vast potential of LLMs, particularly in neurology, even without neurology-specific pretraining. Potential applications in clinical settings include documentation and decision-making support systems as well as educational tools.^35^ LLM 2 passed a neurology board–style examination after exclusion of video and image questions. As deep learning architectures are continuously refined for computer vision and medical imaging,^36,37^ this image-processing limitation may be addressed in future models, potentially including the upcoming multimodal input functionalities of LLM 2. Despite performing admirably on questions assessing basic knowledge and understanding, the model showed slightly lower performance on higher-order thinking questions. Consequently, neurologists should be aware of these limitations, including the models’ tendency to phrase inaccurate responses confidently, and should be cautious regarding its usage in practice or education. With the anticipated advancements in LLMs, neurologists and experts of other clinical disciplines will need to comprehend their performance, reliability, and applications within neurology better. Investigating the potential applications of LLMs that have been fine-tuned specifically for neurology represents a compelling direction for future research.

## Notes

Supplement 1. **eTable 1.** Performance of LLM 1 and LLM 2 on the EBN Question Samples Cohort **eTable 2.** Comparison of LLM 1, LLM 2, and Question Bank Users by Question Type, Difficulty, and Topic **eFigure 1.** Example of a Correctly Answered Lower-Order Question and Answers by LLM 1 and LLM 2 **eFigure 2.** Example of a Correctly Answered Higher-Order Question and Answers by LLM 1 and LLM 2 **eFigure 3.** Example of an Incorrectly Answered Lower-Order Question and Answers by LLM 1 and LLM 2 **eFigure 4.** Example of an Incorrectly Answered Higher-Order Question and Answers by LLM 1 and LLM 2 **eFigure 5.** Confidence of Language in Correctly and Incorrectly Answered Questions **eFigure 6.** Length of Question Between Incorrectly and Correctly Answered Questions Between LLM 1, LLM 2, and Question Bank Users Separately, Primary vs High-Order Question Percentage **eFigure 7.** High-Dimensional tSNE Analyses of Question and Answer Embeddings
eMethods.

eReferences.

Supplement 2.
Data Sharing Statement
