# Error in Key Points, Abstract, Methods, and Discussion

**DOI:** 10.1001/jamanetworkopen.2024.0194

**Published:** 2024-01-29

In the Original Investigation titled “Performance of Large Language Models on a Neurology Board–Style Examination,”^1^ published on December 7, 2023, the source of the board-style examination questions used in the study has been clarified and the article has been corrected.^1^
