# Supplementary material for: Performance of Large Language Models on a Neurology Board–Style Examination
Source: JAMA Netw Open. 2023 Dec 7;6(12):e2346721. doi: 10.1001/jamanetworkopen.2023.46721 (PMC10704278; doi:10.1001/jamanetworkopen.2023.46721)
Supplement: Supplement 1. — eTable 1. Performance of LLM 1 and LLM 2 on the EBN Question Samples Cohort eTable 2. Comparison of LLM 1, LLM 2, and Question Bank Users by Question Type, Difficulty, and Topic eFigure 1. Example of a Correctly Answered Lower-Order Question and Answers by LLM 1 and LLM 2 eFigure 2. Example of a Correctly Answered Higher-Order Question and Answers by LLM 1 and LLM 2 eFigure 3. Example of an Incorrectly Answered Lower-Order Question and Answers by LLM 1 and LLM 2 eFigure 4. Example of an Incorrectly Answered Higher-Order Question and Answers by LLM 1 and LLM 2 eFigure 5. Confidence of Language in Correctly and Incorrectly Answered Questions eFigure 6. Length of Question Between Incorrectly and Correctly Answered Questions Between LLM 1, LLM 2, and Question Bank Users Separately, Primary vs High-Order Question Percentage eFigure 7. High-Dimensional tSNE Analyses of Question and Answer Embeddings eMethods. eReferences. [file jamanetwopen-e2346721-s001.pdf]

Supplemental Online Content

Schubert MC, Wick W, Venkataramani V. Performance of large language models on a neurology board–style examination. *JAMA Netw Open.* 2023;6(12):e2346721. doi:10.1001/jamanetworkopen.2023.46721

eTable 1. Performance of LLM 1 and LLM 2 on the EBN Question Samples Cohort

eTable 2. Comparison of LLM 1, LLM 2 and Question Bank Users by Question Type, Difficulty, and Topic

eFigure 1. Example of a Correctly Answered Lower-Order Question and Answers by LLM 1 and LLM 2

eFigure 2. Example of a Correctly Answered Higher-Order Question and Answers by LLM 1 and LLM 2

eFigure 3. Example of an Incorrectly Answered Lower-Order Question and Answers by LLM 1 and LLM 2

eFigure 4. Example of an Incorrectly Answered Higher-Order Question and Answers by LLM 1 and LLM 2

eFigure 5. Confidence of Language in Correctly and Incorrectly Answered Questions

eFigure 6. Length of Question Between Incorrectly and Correctly Answered Questions Between LLM 1, LLM 2, and Question Bank Users Separately, Primary vs High-Order Question Percentage

eFigure 7. High-Dimensional tSNE Analyses of Question and Answer Embeddings

eMethods.

eReferences.

This supplemental material has been provided by the authors to give readers additional information about their work.

© 2023 Schubert MC et al. *JAMA Network Open.*

eTable 1. Performance of LLM 1 and LLM 2 on the EBN Question Samples Cohort

Question Type	Questions N	GPT-3·5 Correct N (%)	LLM 2 Correct N (%)	Adj P Value GPT-3·5 vs LLM 2
**All Questions**	19	10 (52.6)	14 (73.7)	.31
**Order of thinking**				
**Higher**	14	7 (50)	11 (78.6)	.47
**Lower**	5	3 (60)	3 (60)	>.99

Chi-squared test was used to calculate p-values. P-values were adjusted for multiple testing using the *Bonferroni* correction.

© 2023 Schubert MC et al. *JAMA Network Open.*

eTable 2. Comparison of LLM 1, LLM 2 and Question Bank Users by Question Type, Difficulty, and Topic

Order of Thinking	Questions N	Human Correct Mean %	LLM 1 Correct N (%)	LLM 2 Correct N (%)	P Value LLM 1 vs Human	P Value LLM 2 vs Human	P Value LLM 1 vs LLM 2
**Easy Questions (1st quartile)**							
**Higher**	283	93.3	234 (82.7)	275 (97.2)	<.001	.39	<.001
**Lower**	226	92.9	199 (88.1)	214 (94.7)	.87	>.99	.15
**Intermediate Questions (2nd quartile)**							
**Higher**	271	82.1	189 (69.7)	251 (92.6)	.01	.002	<.001
**Lower**	237	82	193 (81.4)	225 (94.9)	>.99	<.001	<.001
**Advanced Questions (3rd quartile)**							
**Higher**	247	69.6	134 (54.3)	193 (78.1)	.005	.32	<.001
**Lower**	207	69.9	145 (70)	188 (90.8)	>.99	<.001	<.001
**Difficult Questions (4th quartile)**							
**Higher**	262	48.5	110 (42)	153 (58.4)	>.99	.23	.002
**Lower**	223	48.5	102 (45.7)	163 (73.1)	>.99	<.001	<.001

Chi-squared test was used to calculate p-values. P-values were adjusted for multiple testing using the *Bonferroni* correction.

© 2023 Schubert MC et al. *JAMA Network Open.*

eFigure 1. Example of a Correctly Answered Lower-Order Question and Answers by LLM 1 and LLM 2

**Figure 1: Example of a lower-order question, answered correctly**	
**Question**	Explanation by LLM 2
**What kind of identification inability does prosopagnosia reflect?**<br><br>**A: Facial expression.**<br>**<u>B: Familiar faces.</u>**<br>**C: Melody in language.**<br>**D: Non-musical sounds.**<br>**E: Pitch of a melody.**	B: Familiar faces<br>Prosopagnosia, also known as face blindness, is a cognitive disorder that affects the ability to recognize familiar faces, including one's own face (self-recognition). It does not affect a person's ability to recognize and interpret facial expressions, sounds, or melodies. It is typically associated with damage to the right fusiform gyrus, a region of the brain that is believed to be critical for face perception.

© 2023 Schubert MC et al. *JAMA Network Open.*

eFigure 2. Example of a Correctly Answered Higher-Order Question and Answers by LLM 1 and LLM 2

Figure 2: Example of a higher-order question, answered correctly	
**Question**	Explanation by LLM 2
**A 51-year-old male, with a history of herpetic shingles in dermatomes T7-10 three years ago complains about pain in the back side of his right leg. He mentioned that there has been some red discoloration of the skin on his right lower leg, but definitely no shingles. On physical examination, he demonstrated normal tone and full strength in all muscle groups bilaterally; reflexes were normal apart from the knee-reflex being somewhat decreased on the right; the adductor reflexes were symmetric. MRI of the lumbal spine showed degenerative disc disease at all lumbar levels, with slight bilateral foraminal stenosis at L3 and L4, but no herniated disc. Results of an electromyogram were normal. His cerebrospinal fluid showed a predominantly lymphocytic pleocytosis (110 cells/L), a raised protein level (570mg/L) and intrathecal Ig-synthesis. Which diagnosis most likely explains the clinical features of this patient?**<br><br>**<u>A: Neuroborreliosis.</u>**<br>**B: Neurosarcoidosis.**<br>**C: Inflammatory plexopathy.**<br>**D: Postherpetic neuralgia.**<br>**E: Wartenberg syndrome.**	A: Neuroborreliosis.<br>Neuroborreliosis is a complication of Lyme disease (caused by the bacterium Borrelia burgdorferi), where the bacteria infect the nervous system. The features of this case that suggest neuroborreliosis include the pain in the right leg, the lymphocytic pleocytosis and raised protein level in the cerebrospinal fluid, and the evidence of intrathecal Ig-synthesis, which indicates an immune response within the central nervous system.<br>Postherpetic neuralgia is a complication of shingles and would typically present with pain in the area of the previous shingles rash, which does not appear to be the case here. Neurosarcoidosis, inflammatory plexopathy, and Wartenberg syndrome would likely present with different clinical and diagnostic findings.<br>It's worth noting that Lyme disease is often associated with a characteristic "bull's-eye" rash (erythema migrans), but it may not appear in all cases, and the disease can still progress to neuroborreliosis without it.<br>Further diagnostic testing, such as serological tests for antibodies against Borrelia burgdorferi, would be needed to confirm the diagnosis.

© 2023 Schubert MC et al. *JAMA Network Open.*

eFigure 3. Example of an Incorrectly Answered Lower-Order Question and Answers by LLM 1 and LLM 2

**Figure 3: Example of a lower-order question, answered incorrectly**	
**Question**	Explanation by LLM 2
**Which of the following symptoms is found with increased frequency in patients with neuropathic pain as compared with patients with nociceptive pain?**<br><br>**A: Deep aching pain.**<br>**B: Lancinating pain.**<br>**C: Pain evoked by local pressure.**<br>**D: Pain on exercise.**<br>**<u>E: Sensation of pins and needles.</u>**	B: Lancinating pain.<br>Lancinating pain is a symptom that is found with increased frequency in patients with neuropathic pain as compared with patients with nociceptive pain. This type of pain is characterized by the sudden, sharp, electric shock-like sensation. The other options may also be experienced by patients with neuropathic pain, but they are not specific to this type of pain.

© 2023 Schubert MC et al. *JAMA Network Open.*

eFigure 4. Example of an Incorrectly Answered Higher-Order Question and Answers by LLM 1 and LLM 2

**Figure 4: Example of a higher-order question, correctly**	
**Question**	Explanation by LLM 2
**A 35-year-old male was involved in a minor car accident when returning home after a tennis match, and attended hospital because of not being aware of objects on his left side. There was no head injury but nevertheless he felt a throbbing right occipital headache, with no other symptoms. On clinical examination, two hours after the accident, there was a left hemianopia. Eye movements and pupillary function were normal. Blood pressure was 160/95 mmHg. No abnormalities were found on a cranial CT-scan. He reported previous attacks of severe unilateral pulsatile headache with nausea and photophobia between the ages of 15 and 35 years. The frequency of headache was about once in 6 months, with a duration of up to 24 hours. Which diagnosis applies best to this case?**<br><br>**A: Internal carotid artery stenosis.**<br>**B: Migrainous infarction.**<br>**C: Reversible vasoconstriction syndrome.**<br>**D: Posterior reversible encephalopathy syndrome.**<br>**<u>E: Vertebral artery dissection.</u>**	B: Migrainous infarction.<br>The term "migrainous infarction" describes a rare type of ischemic stroke that occurs during a migraine with aura. Given this patient's history of severe, unilateral, pulsatile headaches with nausea and photophobia (typical features of migraines), and the current presentation with headache and visual disturbance (hemianopia), migrainous infarction is a plausible diagnosis.<br><br>The onset of visual symptoms after a minor accident could be coincidental, but it's also possible that the stress of the accident triggered a migraine attack. The normal CT-scan findings don't exclude migrainous infarction, as the changes associated with this condition may not be visible on imaging, especially in the early stages.<br><br>The other options (internal carotid artery stenosis, reversible vasoconstriction syndrome, posterior reversible encephalopathy syndrome, and vertebral artery dissection) could potentially cause similar symptoms, but they don't align as closely with this patient's history and presentation. For example, vertebral artery dissection would typically cause symptoms related to the posterior circulation, such as vertigo, imbalance, or difficulty speaking or swallowing, which this patient doesn't have.

© 2023 Schubert MC et al. *JAMA Network Open.*

eFigure 5: Confidence of Language in Correctly and Incorrectly Answered Questions

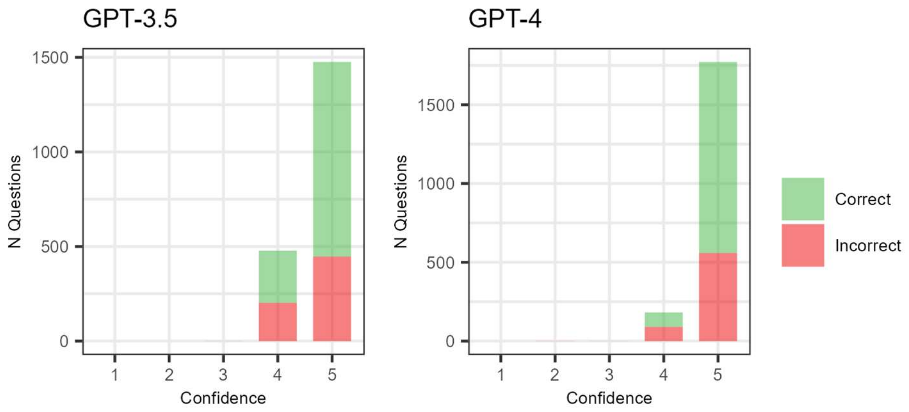

Bar plot visualizing self-assessed confidence on a Likert scale by LLM 1 and LLM 2. Questions are colored based on whether they were answered correctly. (N=1956)

© 2023 Schubert MC et al. *JAMA Network Open.*

eFigure 6. Length of Question Between Incorrectly and Correctly Answered Questions Between LLM 1, LLM 2, and Question Bank Users Separately, Primary vs High-Order Question Percentage

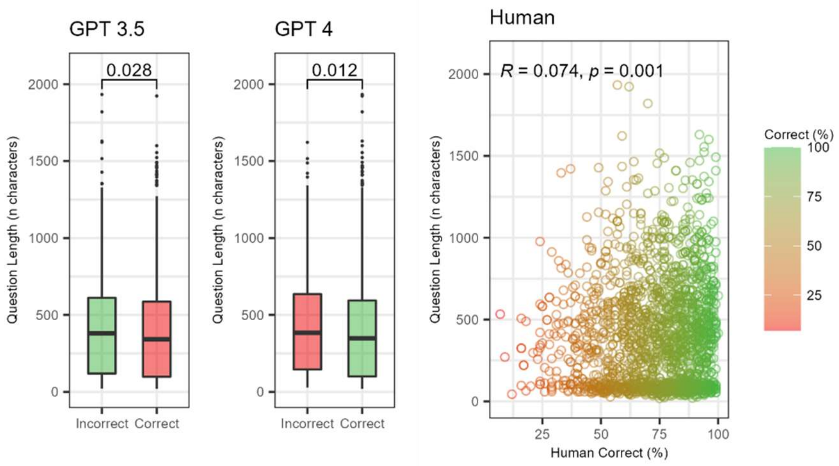

Left: Comparison of Question Length between correctly and incorrectly answered questions for GPT3.5 and GPT4 (N=1956 questions). Right: Correlation plot between the percentage of correctly answering users per question and the question length, one dot representing a single question. Questions are colored based on the perecentage of users correctly answering the question (N=1956).

© 2023 Schubert MC et al. *JAMA Network Open.*

eFigure 7. High-Dimensional tSNE Analyses of Question and Answer Embeddings

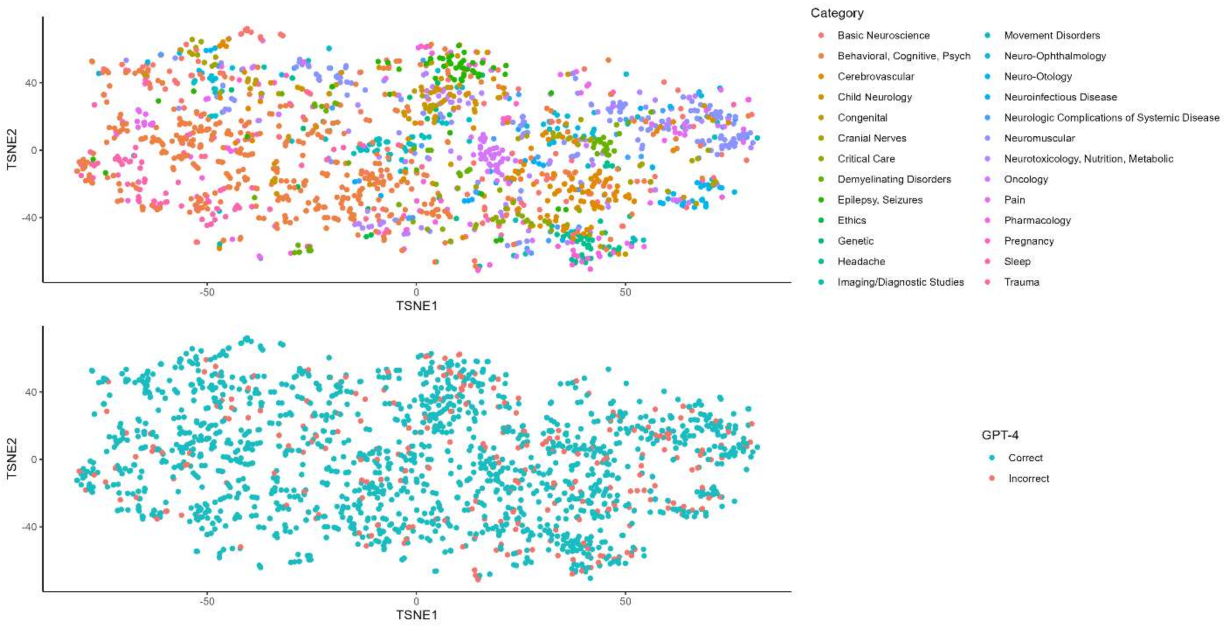

T-SNE analysis of calculated embeddings of questions, each question represented by a single dot and colored based on their related topic.

eMethods.

To test for memorization of the questions and answers, we performed a series of analyses that follow common approaches for analyzing memorization in large language models [1,2]. There, memorization is defined as the "ability to generate the true continuation when choosing the most likely token at every step of decoding" [1]. Tokens are defined as words or groups of characters that appear in a text.

First, in accordance to Carlini et al., we split each multiple-choice problem s in the beginning of the problem a and the true continuation b. We then gave the beginning a to each model to retrieve the model's continuation c, while setting the maximum number of tokens that the model should return to the number of tokens of the true continuation b. To increase the probability to retrieve potential memorizations, we set the model temperature, which is a measure of how deterministic or random the output will be, to 0, to get the most likely and least random continuation.

In both models LLM 1 and LLM 2, in 0 of the 1956 tested multiple choice problems, the beginning a could be correctly continued.

Second, we performed a further analysis, analogous to a method introduced by Biderman et al [2]. For this, a score is defined based on the "number of ordered matching tokens" between the true continuation and the model's continuation c, where the number of matching tokens is divided by the number of tokens in the true continuation. A memorized sequence will have a score equal to 1. In both models LLM 1 and LLM 2, the calculated scores were not equal to 1: (LLM 1: mean = 0.14, SD = 0.1, n = 1956, LLM 2: mean = 0.14, SD = 0.1, n = 1956. As it is known that larger models tend to memorize faster [3], we hypothesized that if the models were trained on the questions, LLM 2 should be able to memorize better than LLM 1. Both models performed equally poor with no significant difference (paired t-test, p=0.64, t-statistic=-0.46, 95 % CI [-0.005, 0.003], n = 1956, mean of the differences: -0.001).

Third, we analyzed the portion of tokens that were matching between the prediction and the true continuation. In LLM 2, the 10 most often matching tokens accounted for 45.46 % of all matching tokens (top 10 matching tokens: " .", " the", " of", " ,", " is", " and", " D", " C", " in", " to"). The occurrence of the tokens " D", " C" is indicative of the model's ability to continue a list if prompted with a beginning string of "A), … B)". Similar results were observed in LLM 1 (top 10 matching tokens: " .", " the", " of", " ,", " is", " and", " C", " D", " in", " patient", representing 46.73% of all matching tokens). The rest of the tokens was composed of a diverse range of terms in the medical context. The complete count tables for the matching tokens for both models are found in Supplementary Material 2.

© 2023 Schubert MC et al. *JAMA Network Open.*

eReferences.

1. Carlini N, Ippolito D, Jagielski M, Lee K, Tramer F, Zhang C. Quantifying Memorization Across Neural Language Models. 2022:arXiv:2202.07646. doi:10.48550/arXiv.2202.07646. Accessed February 01, 2022.
2. Biderman S, Sai Prashanth U, Sutawika L, et al. Emergent and Predictable Memorization in Large Language Models. 2023:arXiv:2304.11158. doi:10.48550/arXiv.2304.11158. Accessed April 01, 2023.
3. Tirumala K, Markosyan AH, Zettlemoyer L, Aghajanyan A. Memorization Without Overfitting: Analyzing the Training Dynamics of Large Language Models. 2022:arXiv:2205.10770. doi:10.48550/arXiv.2205.10770. Accessed May 01, 2022.

© 2023 Schubert MC et al. *JAMA Network Open.*
