# Supplementary material for: Performance of Large Language Models on a Neurology Board–Style Examination
Source: JAMA Netw Open. 2023 Dec 7;6(12):e2346721. doi: 10.1001/jamanetworkopen.2023.46721 (PMC10704278; doi:10.1001/jamanetworkopen.2023.46721)
Supplement: Supplement 2. — Data Sharing Statement [file jamanetwopen-e2346721-s002.pdf]

Data Sharing Statement

Schubert. Performance of Large Language Models on a Neurology Board–Style Examination. *JAMA Netw Open*. Published December 07, 2023. doi:10.1001/jamanetworkopen.2023.46721

Data
Data available: Yes
Data types: Data (not involving human participants)
How to access data: https://github.com/venkataramani-lab/NeurologyBoard_LLM
When available: With publication

Supporting Documents
Document types: Statistical/analytic code
How to access documents: https://github.com/venkataramani-lab/NeurologyBoard_LLM
When available: With publication

Additional Information
Who can access the data: Publicly available data.
Types of analyses: For every purpose.
Mechanisms of data availability: Publicly available without investigator support.
